# Questioning the source of identified non-foodborne pathogens from food-contact wooden surfaces used in Hong Kong's urban wet markets

**DOI:** 10.1016/j.onehlt.2021.100300

**Published:** 2021-08-05

**Authors:** Subramanya Rao, Wing Yui Ngan, Long Chung Chan, Patrick Thabang Sekoai, Aster Hei Yiu Fung, Yang Pu, Yuan Yao, Olivier Habimana

**Affiliations:** The School of Biological Sciences, The University of Hong Kong, Pokfulam, Hong Kong Special Administrative Region

**Keywords:** Hong Kong's Wet market, Wooden cutting board, Non-foodborne pathogens, Phylogenetic comparison

## Abstract

In this study, a phylogenic analysis was performed on pathogens previously identified in Hong Kong wet markets' cutting boards. Phylogenetic comparisons were made between phylotypes obtained in this study and environmental and clinical phylotypes for establishing the possible origin of selected bacterial species isolated from wet market cutting board ecosystems. The results reveal a strong relationship between wet market bacterial assemblages and environmental and clinically relevant phylotypes. However, our poor knowledge of potential cross-contamination sources within these wet markets is further exacerbated by failing to determine the exact or presumed origin of its identified pathogens. In this study, several clinically relevant bacterial pathogens such as *Klebsiella pneumoniae*, *Streptococcus suis* and *Streptococcus porcinus* were linked to cutting boards associated with pork; *Campylobacter fetus*, *Staphylococcus aureus*, *Escherichia coli*, and *A. caviae* in those associated with poultry; and *Streptococcus varanii*, *A. caviae*, *Vibrio fluvialis*, and *Vibrio parahaemolyticus* in those associated with seafood. Identifying non-foodborne clinically relevant pathogens in wet market cutting boards in this study confirms the need for safety approaches for wet market meat, including cold storage. The presented study justifies the need for future systematic epidemiological studies to determine identified microbial pathogens. Such studies should bring about significant improvements in the management of hygienic practices in Hong Kong's wet markets and work towards a One Health goal by recognizing the importance of wet markets as areas interconnecting food processing with animal and clinical environments.

## Graphical abstract

**Figure:**
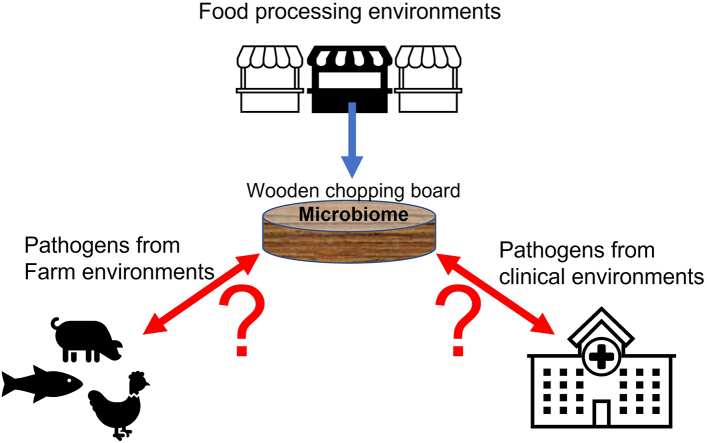

## 1 Introduction

Hong Kong's wet markets continue to be recognized as long-established zones facilitating access to fresh foods. Over the years, thanks to public health awareness, significant efforts have been made towards improving the safety and quality of processed fresh meats in these wet markets [1,2]. Nevertheless, despite the increase in food safety awareness, wet markets have repeatedly been identified as epicenters of potential public health hazards [[3], [4], [5], [6], [7]], primarily biological hazards.

Microbial examination of pork from local wet markets revealed the presence of *Escherichia coli*, molds, and *Salmonella*, indicating the potentially hazardous nature of the meat [1]. Reports have also suggested that pathogens such as *Salmonella* spp., *Staphylococcus aureus*, *Vibrio parahaemolyticus*, and *Listeria monocytogenes* are commonly associated with traditional Chinese processed meats known as Sui-mei and Lo mei [8,9]. Elsewhere, *Laribacter hongkongensis* have been linked with community-acquired gastroenteritis and travelers' diarrhea from minced freshwater fish meat [10]. Furthermore, skin injuries, such as cuts, during meat preparation have been shown to be potential entrance points for pathogens such as *Streptococcus suis* and *Streptococcus iniae* [[11], [12], [13]]. In recent years Hong Kong has witnessed noticeable increases in *Vibrio parahaemolyticus* food poisoning cases [14].

Wet markets are densely populated hubs characterized by a large influx of customers and regulated or unregulated meats, and the hygiene level of the wooden cutting boards used to process these meats remains poorly described. Previous reports from Hong Kong wet markets noted a significant breach in cleaning standards meant for wooden cutting boards, in which surface scraping was used in most studied cases as a traditional cleaning technique [5,15]. Further analyses revealed that these hygienic practices were incapable of guaranteeing proper surface hygiene. Clinically relevant species such as *Klebsiella pneumoniae* exhibiting potential resistance to an array of multiple antibiotics were isolated and identified among microbial communities found on wet market cutting boards [15].

It has been previously established that the improper hygienic maintenance of wooden cutting boards can lead to the development of biofilm niches within their cracked surface patterns [16,17]. Biofilm formation dynamics can be summarized by the initial reversible and irreversible attachment of planktonic cells when first interacting with the abiotic surface, followed by a consolidation stage where, under ideal growing conditions, the adhered cells form microcolonies. The establishment of macrocolonies characterizes the last stage of the biofilm formation dynamic, otherwise recognized as a mature biofilm [18]. Biofilm detachment can arise at different stages of the biofilm formation dynamic. In the case of the surface microcosm of cutting boards, it may lead to the release and transfer of bacterial cells onto the foods being processed [19]. The wooden cutting board surface can be described as a porous material with hydrophilic properties that can provide a suitable environment conducive to the harboring, persistence and proliferation of spoilage and diverse pathogenic organisms [9,20,21]. The processing of raw meat on cutting boards usually leaves behind an abundance of nutrients on its surface, allowing for the proliferation of microbial contaminants [22,23], consequently increasing the likelihood of spreading disease-causing microorganisms when hygiene standards are not met. Therefore, a failure to properly clean cutting boards may promote further biofilm formation, especially the development of the biofilms' most crucial attribute, its matrix of extracellular polymeric substances. This biofilm matrix, synthesized by the cells embedded within the biofilm, acts as a protective barrier against antimicrobial agents and a nutrient trap, allowing for the survival and persistence of embedded cells [[24], [25], [26], [27]]. Cells are well protected in this matrix, including a wide range of microbial pathogens.

A recent microbial profiling study revealed significant differences in hygienic cleaning protocols and access to modern meat processing facilities in 11 Hong Kong wet markets [5]. That study demonstrated that that inefficient hygienic routine practices of cutting boards were responsible for harboring foodborne pathogenic organisms belonging to *Campylobacter*, *Clostridium*, *Escherichia*, *Staphylococcus*, and *Vibrio genera*. Moreover, other pathogen species such as *Klebsiella pneumoniae*, *Enterobacter cloacae*, and *Vibrio vulnificus*, known for causing nosocomial infections, were also found repeatedly on these same cutting boards [5].

Despite these findings, the exact source of the detected biological hazards remains unclear. Pinpointing the likely source would help clarify possible contamination paths, thereby potentially improving existing hygienic routines and cross-contamination measures via improvements in food safety regulations and public health policies. From a One Health perspective, Hong Kong wet markets can therefore be described as a hub in which multiple contamination sources could merge during food processing, ultimately leading to the potential spread of biological contaminants. Although a recent study showed that wet market cutting board hygiene factors affected the prevalence of non-foodborne pathogens on the cutting board, our study sought to validate further the origins of identified non-foodborne pathogens via phylogenic analyses. Here, the full-length 16S ribosomal RNA gene sequences of pathogens identified on cutting boards used in the processing of pork, poultry, and seafood in various wet markets in Hong Kong were used to construct a phylogenetic tree with global datasets on foodborne vs clinically relevant pathogens.

## 2 Methods

### 2.1 Study area and sample collections

Samples were previously obtained from traditional or modern wet markets [5]. Traditional wet markets are located outdoors or in indoor environments without air conditioning. Modern wet markets generally have operational air-conditioning systems and are typically located in buildings meant for wet market activities. The exact wet market sampling locations were presented by Ngan et al. (2020) [5]. In these markets, swab samples were taken in July 2019 from wooden cutting boards meant for pork, poultry, and seafood processing.

Environmental swabs (Zymo, CA, U.S.A.) were sampled from an area of approximately 18 × 8 cm on the boards, as previously described by Lo et al. (2019) [15], with slight modifications. The swab samples were preserved in DNA/RNA shield collection tubes (R1107, Zymo), allowing the preservation of sampled DNA for up to 1 year at room temperature. For each sample, the total genomic DNA (gDNA) was extracted within one month of sampling. DNA extraction and sequencing were performed as previously described by Ngan et al. (2020) [5].

### 2.2 Screening of pathogens from wet market cutting boards

The Divisive Amplicon Denoising Algorithm (DADA2) [28] was used to infer amplicon sequence variants (ASVs) that differed from each other at least by a single nucleotide. The ASVs were inferred from filtered reads obtained by the new version 1.12.1 of the DADA2 R-software package. The latest version had previously been updated for the efficient processing of long amplicon reads and for appropriately modeling PacBio CCS sequencing errors [28].

This study reports 16S full-length rRNA gene phylogeny in samples from wooden cutting boards used to process pork, poultry, and seafood. An earlier study reported the presence of food-associated pathogens [5]. Phylogenetic analysis was conducted to understand the affiliation of these pathogens to clinical isolates; these analyses included 52 ASVs associated with cutting boards used for pork, 17 associated with cutting boards used for poultry, and 13 associated with cutting boards used for seafood.

### 2.3 Phylogenetic analyses

Multiple-sequence alignments for each dataset were conducted using the Muscle program [29] and optimized manually using the Bio-edit program; these data sets were manually edited using Bio-edit [30]. Maximum likelihood trees were estimated using iqtree v0.9.5 [31] using the best-fit nucleotide substitution model [32] chosen by the Bayesian information criterion. An ultrafast bootstrap approximation (UFBoot) was used to assess branch support [31,33]. Here, the number of bootstraps was 1000 replicates. Furthermore, several fast branch tests were carried out using SH-aLRT phylogenetic testing, which was also set to 1000 replicates.

## 3 Results

### 3.1 Meta-analysis of bacterial species composition on wooden cutting boards

**Fig. 1:**
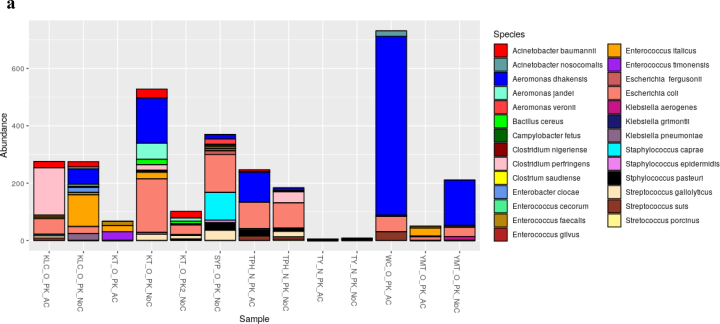

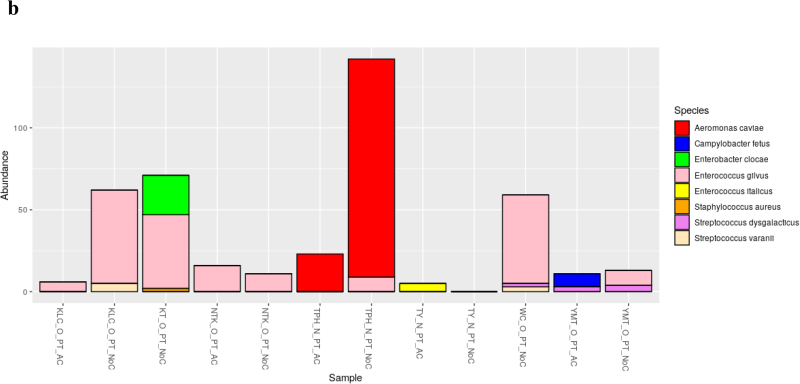

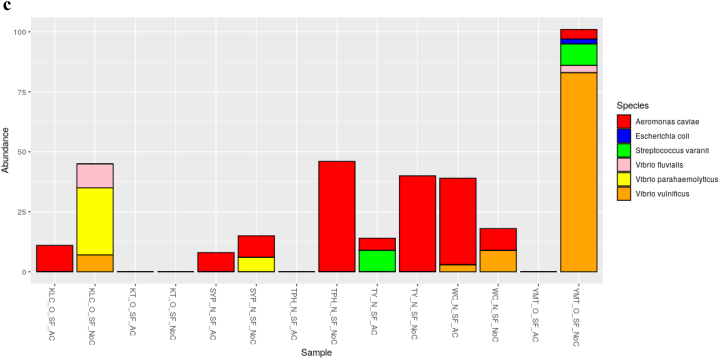
Relative species-level abundance of bacterial pathogens in samples from wooden cutting boards used for different food groups: a) pork, b) poultry, and c) seafood.

The dominant bacterial species in samples from cutting boards used to process pork at wet markets were *Aeromonas dhakensis* and *Escherichia coli* (Fig. 1a). In contrast, those found in samples associated with poultry included *A. caviae* and *Enterococcus gilvus* (Fig. 1b), and those found in samples from seafood cutting boards were *A. caviae*, *Vibrio vulnificus*, and *Vibrio parahaemolyticus* (Fig. 1c). Phylogenetic analysis indicated that the 16S rRNA genes obtained via metagenomic sequencing of wet market wooden cutting board microbiomes were affiliated with clinically relevant human pathogens and food pathogens, predominantly in the Proteobacteria and Firmicutes phyla, respectively.

### 3.2 Phylogeny of identified pathogens associated with cutting boards used to process pork

**Fig. 2:**
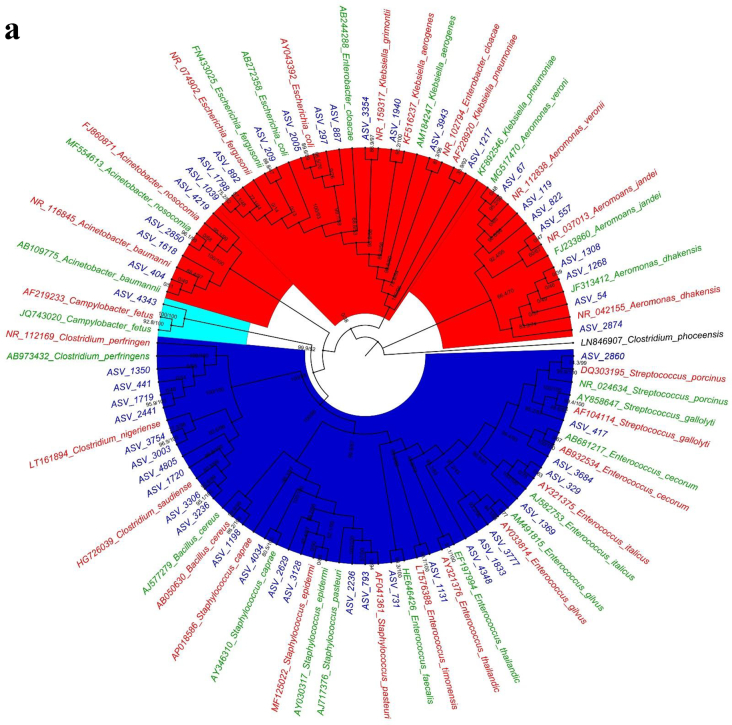

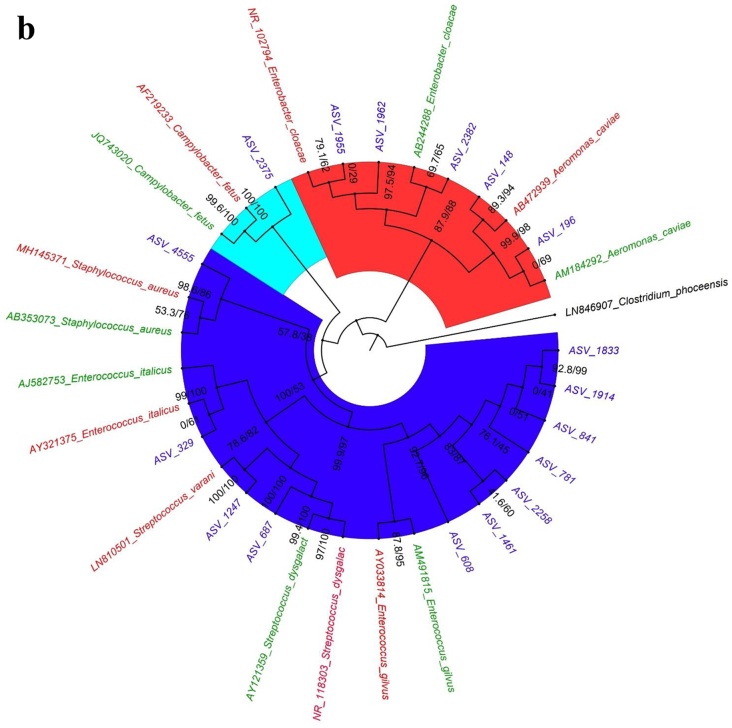

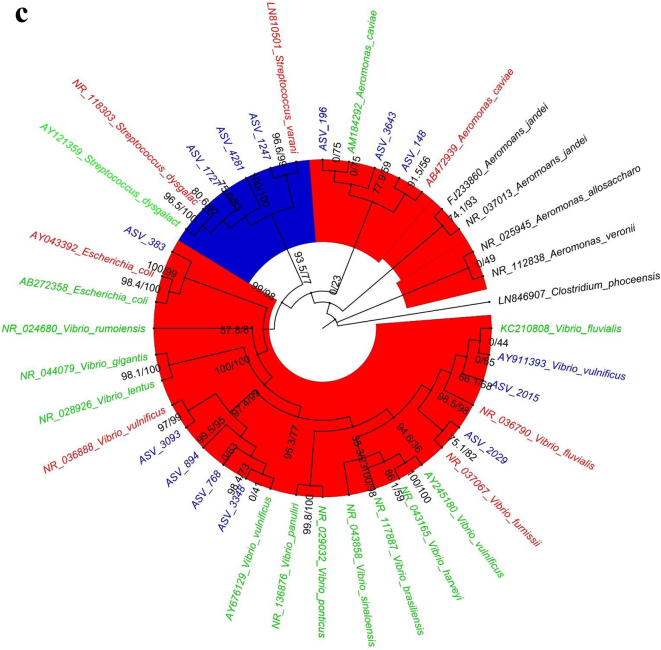
Phylogenetic analysis using Maximum likelihood method for Bacterial pathogens in samples from wooden cutting boards used for different food groups: a) pork, b) poultry, and c) seafood. Blue areas depict *Firmicutes* (phylum)-clade; red areas, *Proteobacteria* (phylum)-clade; and cyan areas, *Campylobacter* (genus)-clade. The green text represents environmental NCBI reference sequences, whereas red text portrays human associated clinical reference sequences. (For interpretation of the references to colour in this figure legend, the reader is referred to the web version of this article.)

The 16S rRNA gene phylogenetic analysis (Fig. 2a) showed that most of the ASVs clustered together with the human-associated clinical strains with high Bootstrap and Bayesian support (Table 1). Thirty-five ASVs from the pork cutting boards clustered closely with the human clinically associated 16S rRNA reference sequences (Table 1), including the following bacterial species: *Aeromonas dhakensis*, *Aeromonas jandei*, *A. veronii*, *Klebsiella pneumoniae*, *Klebsiella grimontii*, *Enterobacter cloacae*, *Escherichia coli*, *Escherichia fergusonii*, *A. nosocomialis*, *A. baumannii*, *Campylobacter fetus*, *Clostridium perfringens*, *Clostridium nigeriense*, *Clostridium saudiense*, *Bacillus cereus*, *Staphylococcus caprae*, *Staphylococcus epidermis*, *Staphylococcus pasteuri*, *Enterococcus faecalis*, *Enterococcus timonensis*, *Enterococcus gilvus*, *Enterococcus italicus*, *Enterococcus cecorum*, *Streptococcus gallolyticus*, and *Streptococcus porcinus*. The remaining 17 ASVs clustered with environment-associated reference sequences, which included the following bacterial species: *A. dhakensis*, *A. jandei*, *A. veronii*, *E. cloacae*, *E. fergusonii*, *A. nosocomialis*, *A. baumannii*, *C. fetus*, *C. nigeriense*, *C. saudiense*, *B. cereus*, *S. epidermis*, *S. pasteuri*, *E. timonensis*, *E. gilvus*, *E. italicus*, *E. cecorum*, *S. gallolyticus*, and *S. porcinus*.

**Table 1:** Comparison of Human/ Environmental association of Bacterial species on wooden cutting board surface used for pork meat processing in Hong Kong wet markets.

Bacterial species	Human associated ASVs	Bootstrap/Bayesian values	Environmental associated ASVs	Bootstrap/Bayesian values
*Aeromonas dhakensis*	1	83.3/74	3	0/49
*Aeromonas jandei*	1	60/67	1	0/47
*A. veronii*	3	95.2/99	1	0/48
*Klebsiella pneumoniae*	1	33.9/92	0	
*Klebsiella aerogenes*	1	96.2/100	0	
*Klebsiella grimontii*	1	86.9/97	0	
*Enterobacter cloacae*	0		1	68.8/53
*Escherichia coli*	3	96.5/76	0	
*Escherichia fergusonii*	3	87.1/48	1	88.8/47
*A. nosocomialis*	1	95.1/99	1	96.1/99
*A. baumannii*	1	88/97	1	0/51
*Campylobacter fetus*	1	100/100	0	
*Clostridium perfringens*	0		4	0/58
*Clostridium nigeriense*	4	77.3/98	0	
*Clostridium saudiense*	2	90.6/99	0	
*Bacillus cereus*	1	86.2/100	0	
*Staphylococcus caprae*	0		1	80.5/100
*Staphylococcus epidermidis*	2	86.7/98	0	
*Staphylococcus pasteuri*	1	0/94	1	100/100
*Enterococcus faecalis*	0		1	98.3/100
*Enterococcus timonensis*	1	99.7/100	0	
*Enterococcus gilvuste*	3	93.2/96	0	
*Enterococcus italicus*	1	0/75	1	100/100
*Enterococcus cecorum*	1	100/100	0	
*Streptococcus gallolyticus*	1	48.6/92	0	
*Streptococcus porcinus*	1	74.3/99	0	

### 3.3 Phylogeny of identified pathogens associated with cutting boards used to process poultry

Phylogenetic analysis of the pathogenic bacteria isolated from wooden cutting boards used for poultry processing showed that they were affiliated with 10 ASVs (Fig. 2b) clustered with human-associated ASVs (Table 2). Clinically relevant species included *A. caviae*, *E. cloacae*, *C. fetus*, *S. aureus*, *E. italicus*, *Streptococcus varanii*, *Streptococcus dysgalactiae*, and *Enterobacter gilvuste*. The remaining seven ASVs were clustered with environmental strains, including *A. caviae*, *E. cloacae*, *S. dysgalactiae*, and *E. gilvuste*.

**Table 2:** Comparison of Human/ Environmental association of Bacterial species on wooden cutting board surface used for poultry meat processing in Hong Kong wet markets.

Bacterial species	Human associated ASVs	Bootstrap/Bayesian values	Environmental associated ASVs	Bootstrap/Bayesian values
*A. caviae*	1	89.3/94	1	0/67
*Enterobacter cloacae*	2	97.5/94	1	69.7/65
*Campylobacter fetus*	1	100/100	0	
*Staphylococcus aureus*	1	98.6/86	0	
*Enterococcus italicus*	1	0/61	0	
*Streptococcus varanii*	1	100/100	0	
*Streptococcus dysgalactiae*	0		1	99.4/100
*Enterobacter gilvuste*	0		7	92.7/96

### 3.4 Phylogeny of identified pathogens associated with cutting boards used to process seafood

For pathogens isolated from cutting boards used to process seafood, the phylogenetic analysis revealed the association of nine ASVs to the human clinical samples (Fig. 2c). The bacterial species included *A. caviae*, *E. coli*, *S. varanii*, *Vibrio furnissii*, *Vibrio fluvialis*, and *Vibrio vulnificus*. The other four ASVs clustered with *A. caviae* and *V. vulnificus* (Table 3).

**Table 3:** Comparison of Human/ Environmental association of Bacterial species on wooden cutting board surface used for seafood meat processing in Hong Kong wet markets.

Bacterial species	Human associated ASVs	Bootstrap/Bayesian values	Environmental associated ASVs	Bootstrap/Bayesian values
*A. caviae*	1	91.5/56	2	0/75
*Streptococcus varanii*	3	96.6/99		
*Escherichia coli*	1	100/99		
*Vibrio vulnificus*	2	99.5/95	2	98.4/73
*Vibrio furnissii*	1	75.1/82		
*Vibrio fluvialis*	1	98.5/98		

## 4 Discussion

This study aimed to characterize the presence of pathogens on cutting boards from Hong Kong's wet markets and determine their presumptive source via phylogenetic analysis. This study represents a complete phylogenetic assessment of wet market foodborne and clinically relevant bacterial pathogens. Both environmental and clinically relevant datasets of 16S rRNA were used to test the phylogenetic affiliations among pathogens from the cutting board samples. In this study, the phylogenetic analysis of the pathogenic bacterial assemblages from cutting boards used to process pork, poultry, and seafood identified a clear association with human-associated and clinically relevant phylotypes.

The porous surfaces of wooden cutting boards are perfect channels for the circulation of nutrients and water and thus provide favorable conditions for biofilm-forming communities. Furthermore, the lack of proper hygienic maintenance of these wooden cutting boards may have led to the establishment of niche-harboring pathogenic bacteria that can form biofilms. In this study, cutting board pathogens were dominated by *Aeromonas dhakensis*, *A. caviae*, *A. jandei*, and *A. veronii*. Earlier reports suggested that *Aeromonas* has the remarkable ability to colonize different environments through biofilm formation and cell-cell signaling [34]. Moreover, *Aeromonas* species were likewise observed in mixed-species in food contact surfaces [35]. *Aeromonas* also plays a significant role in many health conditions, including gastroenteritis, wound infections, bacteremia, and, although less frequently, peritonitis, urinary tract infections, and ocular infections [36]. In this study, *E. coli*, another biofilm-forming pathogen, was linked with cutting boards associated with pork and seafood processing. It is well established that *E. coli* is a commensal organism predominantly associated with the gastrointestinal tract in animals and humans alike, where it thrives in complex biofilm consortia characterized by a plethora of other microorganisms [37,38]. Furthermore, in clinical environments, *E. coli* has also been found to contaminate medical devices such as catheters, leading to catheter-associated urinary tract nosocomial infections [39]. *Enterococcus* was predominantly recovered in cutting boards associated with pork and poultry when assessing other biofilm-forming pathogens, suggesting its persistence in the wet market food processing environment. An earlier report demonstrated enterococci's ability to form biofilms [40]. Elsewhere, in clinical settings, enterococcal biofilms associated with infections are hard to eradicate, given their high tolerance to antimicrobials [41].

The finding of foodborne and non-foodborne pathogens in wet market cutting board settings should be considered an alarming indicator of poor hygienic conditions. Recent studies have indicated that poor hygiene practices at wet markets may have exposed cutting boards to spoilage and pathogenic surface contamination [5,19]. Regular surface hygiene of such wet market cutting boards may be necessary. The food contact surface may interact with previously contaminated foods during processing, especially considering that these wet markets are usually characterized by poor storage/display conditions linked to inappropriate temperature control or lack thereof. The identification of *Streptococcus suis* on cutting boards used to process pork suggests poor storage conditions, leading to its proliferation over time and its transfer to cutting boards during processing (Fig. 1). Earlier reports in Hong Kong have shown that *S. suis* is a key bacterial pathogen responsible for various human infections in Hong Kong [42]. Although *S. suis* is enteric and nonpathogenic in pigs, its spread when handling raw pork products through cross-contamination increases the likelihood and risk of infections [12]. In 2019, the Hong Kong Centre for Health Protection (CHP) investigated an *S. suis* infection in a patient who died following fever, abdominal pain, vomiting, and diarrhea [43]. The initial investigations revealed that the patient had handled raw pig organs during the incubation period. In 2005, Hong Kong temporarily suspended all pork imports from Sichuan due to an *S. suis* outbreak. The Ministry of Health of China once reported 215 cases of human disease associated with the outbreak, 39 of which were fatal [44].

Another streptococcal species associated with various pathological infections in swine, *S. porcinus*, was identified as having potential clinically relevant associations. Earlier reports identified *S. porcinus* in female genitourinary tracts [[45], [46], [47]]. Other pathogens such as *Staphylococcus aureus* in cutting boards used for poultry also show a close affiliation to human-associated clinical phylotypes (Fig. 2). Earlier studies indicated *S. aureus*'s ability to survive, colonize, and persist in poultry processing plants [48]. More specifically, the persistence of *S. aureus* was shown to be linked to its ability to adhere to different types of material [48,49] and to withstand cleaning and disinfection through the synthesis of a glucosamine-rich extracellular polymer [48,49]. Phylogenetic analysis of the cutting board pathogens showed the affiliation of ASVs in cutting boards used for processing pork and poultry meat with clinically relevant *Campylobacter fetus*, a foodborne illness pathogen among humans. *C. fetus* is also known to cause bacteremia and thrombophlebitis [50], and in rarer cases, can cause sepsis in newborn and immunocompromised individuals [51].

Evolutionary analysis of our bacterial 16S rRNA data found that several non-foodborne pathogens identified on cutting boards had a high likelihood of clinical relevance. For instance, *Klebsiella pneumoniae*, isolated from pork cutting boards, had a high affinity to clinically relevant nosocomial pathogens affiliated to a human-associated phylotype (AF228920) isolated from human urine (Fig. 1). This observation may suggest that wet markets in Hong Kong either lack proper sanitary toilets and handwashing stations or have precarious proximity to clinics and hospitals. In the latter case, the closeness of hospitals and wet markets is not unusual in Hong Kong. For example, in Hong Kong's Kowloon district (KLC) (Fig. 1), one wet market was surrounded by three hospitals/clinics (Supplementary Table 1). Regardless of the location, access to handwashing stations and their proper use could reduce the spread of non-foodborne pathogens [52]. The general lack or improper usage of sanitation stations at these wet markets may have led to our finding of other pathogens that are phylogenetically affiliated to clinical strains, including the identified species *A. nosocomialis* and *A. baumannii* in cutting boards used to process pork; *A. caviae* and *Enterobacter cloacae* in cutting boards used to process poultry, and *A. caviae* and *Vibrio parahaemolyticus* in cutting boards used to process seafood. The significance of such non-foodborne pathogens on food processing surfaces should not be ignored because of the possibility of their additional anti-microbial resistance properties. The persistence and survival of most nosocomial pathogenic organisms in hospitalised patients' flora and the surrounding environment can be attributed to their multi-drug resistance abilities [[53], [54], [55]]. Epidemiological approaches in the characterization and tracking of pathogens have allowed for implementing safety and prevention measures for improving public health. In a previous outbreak at the National Institute of Health (USA), an epidemiological investigation helped further our understanding of the spread of *K. pneumoniae* and its ability to increase its antibiotic resistance [56]. Our study lacks the detailed epidemiological data needed to confirm whether these pathogens originate from nearby hospitals or track their source. One of the limitations of studying hospital-associated infection (HAI) is the lack of molecular assays. All HAI confirmations thus far have relied on culturing techniques.

Future work should incorporate different models, including geospatial system models, to evaluate pathogens' true origin in wet markets. Adopting whole genomic-based approaches, the quantification and characterization of identified pathogens by integrating genetic and epidemiological information would systematically improve wet markets' surveillance routines, ultimately strengthening food safety policies.

## 5 Conclusions

This study investigated the phylogenetic relationship among bacterial communities associated with Hong Kong's wet market wooden cutting boards used for meat, poultry, and seafood processing. This was achieved via high-throughput metagenomic sequencing of full-length bacterial 16S rRNA amplicons. The data were then compared with environmental and clinically associated pathogens. First, the pathogens in cutting boards used for pork were more diverse than those used for poultry and seafood. Second, the phylogenetic analysis indicated that the wet market wooden cutting board bacterial communities were closely affiliated to human pathogenic strains associated with clinical infections. Thus, improvements in meat storage conditions are critical to avoid pathogen contamination in wet markets. Furthermore, refrigeration and cooling infrastructure at wet markets would improve the safe storage and display of raw meat. Such installations would delay the growth of unwanted and pathogenic microorganisms and their dissemination into the surrounding environment via cross-contamination. Finally, cleaning and sanitation stations would also help reduce the potential spread of non-foodborne pathogens by improving general personal hygiene.

## Data availability

Raw sequencing reads have been deposited in the EMBL-EBI Sequence Read Archive under the accession number PRJEB37431.

## Declaration of Competing Interest

The authors declare no conflict of interest.

## References

[1] O’Toole D.K. (1995). Microbiological quality of pork meat from local Hong Kong markets. World J. Microbiol. Biotechnol..

[2] Wu X., Ye Y., Hu D., Liu Z., Cao J. (2014). Food safety assurance systems in Hong Kong. Food Control.

[3] Chan J.F., Yuan S., Kok K.H., To K.K., Chu H., Yang J., Xing F., Liu J., Yip C.C., Poon R.W., Tsoi H.W., Lo S.K., Chan K.H., Poon V.K., Chan W.M., Ip J.D., Cai J.P., Cheng V.C., Chen H., Hui C.K., Yuen K.Y. (2020). A familial cluster of pneumonia associated with the 2019 novel coronavirus indicating person-to-person transmission: a study of a family cluster. Lancet.

[4] Lee J.W., McKibbin W.J., Knobler S., Mahmoud A., Lemon S., Oberholtzer K., Sivitz L., Mahmoud A., I.o. Medicine, B.o.G. Health, F.o.M. Threats (2004). Estimating the global economic costs of sars. Learning from SARS: Preparing for the Next Disease Outbreak: Workshop Summary.

[5] Ngan W.Y., Rao S., Chan L.C., Sekoai P.T., Pu Y., Yao Y., Fung A.H.Y., Habimana O. (2020). Impacts of wet market modernization levels and hygiene practices on the microbiome and microbial safety of wooden cutting boards in Hong Kong. Microorganisms.

[6] Xu R.H., He J.F., Evans M.R., Peng G.W., Field H.E., Yu D.W., Lee C.K., Luo H.M., Lin W.S., Lin P., Li L.H., Liang W.J., Lin J.Y., Schnur A. (2004). Epidemiologic clues to SARS origin in China. Emerg. Infect. Dis..

[7] Yuen K.Y., Chan P.K., Peiris M., Tsang D.N., Que T.L., Shortridge K.F., Cheung P.T., To W.K., Ho E.T., Sung R., Cheng A.F. (1998). Clinical features and rapid viral diagnosis of human disease associated with avian influenza A H5N1 virus. Lancet.

[8] CFC, (Centre for Food Safety) (2009). Reference materials in developing food safety plan for selling of Siu Mei and Lo Mei. https://www.cfs.gov.hk/english/programme/programme_haccp/programme_haccp_slmltraining_factors.html.

[9] Engelund E.T., Thygesen L.G., Svensson S., Hill C.A.S. (2013). A critical discussion of the physics of wood–water interactions. Wood Sci. Technol..

[10] Woo P.C., Lau S.K., Teng J.L., Que T.L., Yung R.W., Luk W.K., Lai R.W., Hui W.T., Wong S.S., Yau H.H., Yuen K.Y., L.H.s. group (2004). Association of *Laribacter hongkongensis* in community-acquired gastroenteritis with travel and eating fish: a multicentre case-control study. Lancet.

[11] Huang Y.T., Teng L.J., Ho S.W., Hsueh P.R. (2005). Streptococcus suis infection. J. Microbiol. Immunol. Infect..

[12] Kay R., Cheng A.F., Tse C.Y. (1995). *Streptococcus suis* infection in Hong Kong. QJM.

[13] Lau S.K., Woo P.C., Tse H., Leung K.W., Wong S.S., Yuen K.Y. (2003). Invasive *Streptococcus iniae* infections outside North America. J. Clin. Microbiol..

[14] CFC, (Centre for Food Safety) (2005). Reference materials in Risk assessment studies Report no.20: Vibrio species in seafood. https://www.cfs.gov.hk/english/programme/programme_rafs/programme_rafs_fm_01_02_vss.html.

[15] Lo M.Y., Ngan W.Y., Tsun S.M., Hsing H.L., Lau K.T., Hung H.P., Chan S.L., Lai Y.Y., Yao Y., Pu Y., Habimana O. (2019). A field study into Hong Kong’s wet markets: raised questions into the hygienic maintenance of meat contact surfaces and the dissemination of microorganisms associated with nosocomial infections. Front. Microbiol..

[16] Edelmeyer H. (1984). Clean cutting boards and knives - is this too much to ask of hygiene. Fleischwirtschaft.

[17] Prechter S., Betz M., Cerny G., Wegener G., Windeisen E. (2002). Hygienische Aspekte von Schneidebrettern aus Holz bzw. Kunststoff. Holz als Roh- und Werkstoff.

[18] Li Q., Xia P., Tao Z., Wang S. (2017). Modeling biofilms in water systems with new variables: a review. Water-Sui.

[19] Sekoai P.T., Feng S., Zhou W., Ngan W.Y., Pu Y., Yao Y., Pan J., Habimana O. (2020). Insights into the microbiological safety of wooden cutting boards used for meat processing in Hong Kong’s wet markets: a focus on food-contact surfaces, cross-contamination and the efficacy of traditional hygiene practices. Microorganisms.

[20] Abdul-Mutalib N.A., Amin Nordin S., Osman M., Ishida N., Tashiro K., Sakai K., Tashiro Y., Maeda T., Shirai Y. (2015). Pyrosequencing analysis of microbial community and food-borne bacteria on restaurant cutting boards collected in Seri Kembangan, Malaysia, and their correlation with grades of food premises. Int. J. Food Microbiol..

[21] Adetunji V.O., Isola T.O. (2011). Crystal violet binding assay for assessment of biofilm formation by *Listeria monocytogenes* and *Listeria* spp on wood, steel and glass surfaces. Global Vet..

[22] An Y.H., Friedman R.J. (1998). Concise review of mechanisms of bacterial adhesion to biomaterial surfaces. J. Biomed. Mater. Res..

[23] Carpentier B. (1997). Sanitary quality of meat chopping board surfaces: a bibliographical study. Food Microbiol..

[24] Flemming H.C., Neu T.R., Wozniak D.J. (2007). The EPS matrix: the “house of biofilm cells”. J. Bacteriol..

[25] Flemming H.C., Wingender J. (2001). Relevance of microbial extracellular polymeric substances (EPSs)--part I: structural and ecological aspects. Water Sci. Technol..

[26] Stewart P.S. (2001). Multicellular resistance: biofilms. Trends Microbiol..

[27] Stewart P.S. (2003). Diffusion in biofilms. J. Bacteriol..

[28] Callahan B.J., Wong J., Heiner C., Oh S., Theriot C.M., Gulati A.S., McGill S.K., Dougherty M.K. (2019). High-throughput amplicon sequencing of the full-length 16S rRNA gene with single-nucleotide resolution. Nucleic Acids Res..

[29] Edgar R.C. (2004). MUSCLE: multiple sequence alignment with high accuracy and high throughput. Nucleic Acids Res..

[30] Hall T.A. (1999). BioEdit: a user-friendly biological sequence alignment editor and analysis program for windows 95/98/NT. Nucleic Acids Symp. Ser..

[31] Nguyen L.T., Schmidt H.A., von Haeseler A., Minh B.Q. (2015). IQ-TREE: a fast and effective stochastic algorithm for estimating maximum-likelihood phylogenies. Mol. Biol. Evol..

[32] Kalyaanamoorthy S., Minh B.Q., Wong T.K.F., von Haeseler A., Jermiin L.S. (2017). ModelFinder: fast model selection for accurate phylogenetic estimates. Nat. Methods.

[33] Wang H.C., Minh B.Q., Susko E., Roger A.J. (2018). Modeling site heterogeneity with posterior mean site frequency profiles accelerates accurate phylogenomic estimation. Syst. Biol..

[34] Talagrand-Reboul E., Roger F., Kimper J.L., Colston S.M., Graf J., Latif-Eugenin F., Figueras M.J., Petit F., Marchandin H., Jumas-Bilak E., Lamy B. (2017). Delineation of taxonomic species within complex of species: *Aeromonas media* and related species as a test case. Front. Microbiol..

[35] Gunduz G.T., Tuncel G. (2006). Biofilm formation in an ice cream plant. Antonie Van Leeuwenhoek.

[36] Janda J.M., Abbott S.L. (2010). The genus Aeromonas: taxonomy, pathogenicity, and infection. Clin. Microbiol. Rev..

[37] Costerton J.W., Lewandowski Z., Caldwell D.E., Korber D.R., Lappin-Scott H.M. (1995). Microbial biofilms. Annu. Rev. Microbiol..

[38] Probert H.M., Gibson G.R. (2002). Bacterial biofilms in the human gastrointestinal tract. Curr. Issues Intest. Microbiol..

[39] Reisner A., Maierl M., Jorger M., Krause R., Berger D., Haid A., Tesic D., Zechner E.L. (2014). Type 1 fimbriae contribute to catheter-associated urinary tract infections caused by *Escherichia coli*. J. Bacteriol..

[40] Hashem Y.A., Amin H.M., Essam T.M., Yassin A.S., Aziz R.K. (2017). Biofilm formation in enterococci: genotype-phenotype correlations and inhibition by vancomycin. Sci. Rep..

[41] Ch'ng J.H., Chong K.K.L., Lam L.N., Wong J.J., Kline K.A. (2019). Biofilm-associated infection by enterococci. Nat. Rev. Microbiol..

[42] Centre for Health Protection (CHP) (2005). Scientific Committee on Emerging and Zoonotic Diseases. *Streptococcus suis* infection in Hong Kong.

[43] CFC, (Center for Food Safety) (2019). *Streptococcus suis* Infection. https://www.chp.gov.hk/en/healthtopics/content/24/3648.html.

[44] Yu H., Jing H., Chen Z., Zheng H., Zhu X., Wang H., Wang S., Liu L., Zu R., Luo L., Xiang N., Liu H., Liu X., Shu Y., Lee S.S., Chuang S.K., Wang Y., Xu J., Yang W. (2006). *Streptococcus suis* study, Human *Streptococcus suis* outbreak, Sichuan, China. Emerg. Infect. Dis..

[45] Duarte R.S., Barros R.R., Facklam R.R., Teixeira L.M. (2005). Phenotypic and genotypic characteristics of Streptococcus porcinus isolated from human sources. J. Clin. Microbiol..

[46] Facklam R.R., Washington J.A., Balows A., Haus-ler W.J., Herrmann K.L., Isenberg H.D., Shadomy H.J. (1991). Manual of clinical microbiology. Streptococcus and Related Catalase-Negative Gram-Positive Cocci.

[47] Martin C., Fermeaux V., Eyraud J.L., Aubard Y. (2004). *Streptococcus porcinus* as a cause of spontaneous preterm human stillbirth. J. Clin. Microbiol..

[48] Chaffey B., Waites W. (1987). The adhesion of Staphylococcus-aureus isolates from poultry-processing plants. J. Appl. Bacteriol.

[49] Mead G.C., Dodd C.E. (1990). Incidence, origin and significance of staphylococci on processed poultry. Soc. Appl. Bacteriol. Symp. Ser..

[50] Perez-Perez G.I., Blaser M.J., Baron S. (1996). Campylobacter and Helicobacter. Medical Microbiology.

[51] Monno R., Rendina M., Ceci G., Rizzo C., Luzzi I., Francavilla A., Rizzo G., Ierardi E. (2004). Campylobacter fetus bacteremia in an immunocompromised patient: case report and review of the literature. New Microbiol..

[52] Bloomfield S.F., Aiello A.E., Cookson B., O'Boyle C., Larson E.L. (2007). The effectiveness of hand hygiene procedures in reducing the risks of infections in home and community settings including handwashing and alcohol-based hand sanitizers. Am. J. Infect. Control.

[53] Asensio A., Oliver A., Gonzalez-Diego P., Baquero F., Perez-Diaz J.C., Ros P., Cobo J., Palacios M., Lasheras D., Canton R. (2000). Outbreak of a multiresistant *Klebsiella pneumoniae* strain in an intensive care unit: antibiotic use as risk factor for colonization and infection. Clin. Infect. Dis..

[54] Jarvis W.R., Munn V.P., Highsmith A.K., Culver D.H., Hughes J.M. (1985). The epidemiology of nosocomial infections caused by *Klebsiella pneumoniae*. Infect. Control..

[55] Pena C., Pujol M., Ardanuy C., Ricart A., Pallares R., Linares J., Ariza J., Gudiol F. (1998). Epidemiology and successful control of a large outbreak due to *Klebsiella pneumoniae* producing extended-spectrum beta-lactamases. Antimicrob. Agents Chemother..

[56] Sandora T.J., Goldmann D.A. (23, 2012). Preventing lethal hospital outbreaks of antibiotic-resistant bacteria. N. Engl. J. Med..

